# Evaluation of Poorly Soluble Drugs’ Dissolution Rate by Laser Scattering in Different Water Isotopologues

**DOI:** 10.3390/molecules26030601

**Published:** 2021-01-24

**Authors:** Elena V. Uspenskaya, Tatiana V. Pleteneva, Ilaha V. Kazimova, Anton V. Syroeshkin

**Affiliations:** Department of Pharmaceutical and Toxicological Chemistry, Medical Institute, RUDN University, 6 Miklukho-Maklaya Street, 117198 Moscow, Russia; pleteneva_tv@pfur.ru (T.V.P.); 1042205160@rudn.university (I.V.K.); syroeshkin_av@pfur.ru (A.V.S.)

**Keywords:** kinetic isotope effect (KIEs), poorly soluble drugs, laser diffraction spectroscopy, dissolution rate, validation studies

## Abstract

The most important task in the design of dosage forms is to modify the pharmaceutical substances structure in order to increase solubilization, targeted delivery, controlled rate of drug administration, and its bioavailability. Screening—laboratory (in vitro) or computer (in silico)—as a procedure for selecting a prototype for the design of a drug molecule, involves several years of research and significant costs. Among a large number of solvents and diluents (alcohol, ether, oils, glycerol, Vaseline) used in the pharmaceutical industry for the manufacture of drugs water finds the greatest application. This is because all biological reactions (reactions in living systems) take place in water and distribution of the fluid in the body and the substances found within is critical for the maintenance of intracellular and extracellular functions. Modern studies in the field of the stable isotopic compositions of natural water and its structure and properties make it possible to use isotopic transformations of the water to improve the pharmacokinetic properties of medicinal substances without previous structural modification. It is known that by replacing any of the atoms in the reacting substance molecule with its isotope, it is possible to record changes in the reactivity, which are expressed as a change in the reaction rate constant, i.e., in the manifestation of the kinetic isotope effect (KIE). The article presents the results of studies on the effect of the kinetic isotope effect of a solvent—water—on increasing the solubility and dissolution rate constants of poorly soluble drugs using laser diffraction spectroscopy. The results of the studies can be successfully implemented in pharmaceutical practice to overcome the poor solubility of medicinal substances of classes II and IV, according to the biopharmaceutical classification system (BCS), in water for pharmaceutical purposes by performing its preliminary and safe isotopic modification.

## 1. Introduction

The solubility characteristics of 40–70% of new drug candidates are so poor that they cannot be formulated on their own and new methods for increasing drug solubility in water are highly prized [1,2]. The major challenge with the design of oral dosage forms lies in their poor bioavailability. The oral bioavailability depends on several factors including aqueous solubility, drug permeability, dissolution rate, first-pass metabolism, presystemic metabolism, and susceptibility to efflux mechanisms. The most frequent causes of low oral bioavailability are attributed to poor solubility and low permeability [3,4]. Here, we describe a new promising method to enhance the solubility of poorly water soluble drugs by using the laser diffraction spectroscopy technique. Current trends in the search for new drug candidates by pharmaceutical companies include the development of systems for targeted drug transport to organs and tissue.However, the chemical and physical properties of their forms need to be modified and optimized for in vivo performance [5,6]. Currently, when developing new active pharmaceutical ingredients, technologies are used to improve their pharmacological properties. This can be conducted by developing a method for targeted delivery of active pharmaceutical ingredients, as well as modifying the absorption rate and the time of drug action. To improve the parameters of drug solubility and absorption, various technological approaches are used, including micronization, salt formation, preparation of amorphous solid dispersions, cocrystals, solubilization in surfactant micelles, lipid-based formulations, and colloidal delivery systems [7].

There are in silico approaches that allow predicting the solubility of compounds based on determining the relative energy difference (RED) of a molecule as a combination of dispersion forces, hydrogen bonds, and energy of the dipolar intermolecular force [8]. Such approaches include, for example, the Flory-Huggins theory, the Hildebrand and Hansen solubility parameters (HSPs) proposed in 1967 [9]. However, these theoretical concepts have a number of limitations [10].

Water is the solvent of choice for liquid pharmaceutical formulations. The water molecule is a unique chemical compound [11]. The formation of a network of intermolecular hydrogen bonds is determined by the interaction between a positively polarized hydrogen atom in one molecule and a negatively polarized oxygen atom in another molecule. The extensive hydrogen bonding provides water with the ability to transfer protons and electrons rapidly between the water molecules and to produce positively charged hydrogen ions and negatively charged hydroxide ions. In many solid phases, each water molecule forms exactly four hydrogen bonds in a tetrahedral arrangement with two hydrogen atoms near each oxygen atom. This arrangement is also present, if much more loosely, in liquid water, where the overall structuring is far more complex. Such liquid water behaves as though it is a mixture of two liquids that change relative composition with variations in temperature and pressure [12,13]. This, in turn, leads to the appearance of many anomalous properties of water and also explains its ability to act as a universal solvent [14,15,16].

### 1.1. Water Isotopologues

Natural water is a multi-component mixture of molecules of different isotopic compositions (isotopologues). Changes in the isotopic composition of substances cause changes in their physical and chemical properties—isotope effects. The isotope effects are relatively high for the hydrogen atom and much lower for the isotopes of other elements. Natural hydrogen is a mixture of isotopes: H11, H12, H13. The nuclei of protium and deuterium are stable, in contrast to radioactive tritium with a half-life of 12.26 years. The quantitative ratios between isotopes of hydrogen are presented as 1:1, 46 × 10^−5^: 4.0·× 10^−15^ [17]. Differences in physical properties of water H211O and H212O cause abnormally high natural variations in the isotopic composition: ^1^H_2_^16^O, ^1^H_2_^17^O, ^1^H_2_^18^O, ^1^HD^16^O, ^1^HD^17^O, ^1^HD^18^O, D_2_^16^O, D_2_^17^O, D_2_^18^O [18]. The study of the effect of hydrogen substitution by its isotopes in water leads to significant changes in the physical constants while maintaining the chemical properties and structure unchanged [19]. In our previous research, we successfully presented experimental results of physical, chemical, and biological effects of water, depleted by heavy isotopes, at various levels of reaction system organization [20,21,22,23]. This opens up new opportunities for studying the physical chemistry of aqueous solutions, including the practical aspect—for alternatives to existing approaches and methods of increasing the thermodynamic solubility and the rate of dissolution and bioavailability of drugs [24].

According to [25,26], direct comparison of reaction rates with different isotopic molecules is not only difficult and inaccurate but often impossible. In connection to this, this work is based on the method of laser diffraction spectroscopy for the development, validation, and evaluation of the kinetic isotope effect in water when dissolving poorly soluble drugs.

#### Selection of Solvent—Water with Lower Deuterium Content than Natural Water (Deuterium-Depleted Water)

It is known that the basis of differences in the properties of water isotopologues consists in the difference in the structure of atoms of the same element—hydrogen, characterized by equal charges in the nucleus and structure of electron shells but different nuclear masses [27]. According to [28], water isotopologues differ from each other in physical and chemical properties and biological effects on living objects of different hierarchical levels. The two-fold difference in the mass of (2)H (deuterium) and (1)H (protium) atoms is manifested in higher activation energy required to reach the transition state when breaking the C–D bond than for C–H bond [29]. This is why the reaction rate with the participation of isotopologues containing an atom of a “light” element is higher than for its deuterium analogue (k_H_ > k_D_) [30,31]. In protium water H211O, the rate and mutarotation of carbohydrates and the optical properties of chiral compounds change, the absorption and accumulation of essential trace element ions in medicinal and food plants increase, and the condition of patients with malignancies improves after using protium water as an adjuvant [32,33,34,35,36]. Shortly after the discovery of stable isotopes of hydrogen, oxygen, and carbon, Jacob Bigeleisen formulated a theory of isotope effects and calculated possible maximum values. Large isotope effects of (2)H (deuterium) against (1)H (protium) were seen to possibly influence interpretations of reaction mechanisms if corresponding labeling was used [37]. Water with a modified hydrogen isotopic composition has physical and chemical features, such as the temperature of phase transitions, surface tension, and others, which lead to significant changes in the properties of aqueous solutions [38].

Thus, the purpose of this work was to study the mechanism of kinetic isotope effect development, which is associated with a change in the D/H ratio in water to control the dissolution process of poorly soluble drugs.

## 2. Results

### 2.1. Low-Angle Static and Dynamic Light Scattering in the Analysis of Dispersion Properties of Water Isotopologues

By measuring the low-angle indicatrix, we determined the parameters of low-angle laser light scattering (LALLS) by the cluster surface in water samples with different contents of the heavy hydrogen isotope (Table 1).

According to Table 1, the depletion and the enrichment of water by the heavy hydrogen isotope leads to a decrease in the light scattering intensity due to a small volume occupied by the water clusters formed.

**Figure 1:**
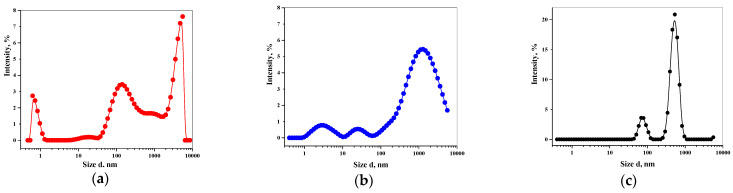
Distribution of water supramolecular complexes by size (DLS method data) in, deuterium-depleted water 1 ppm (**a**), MiliQ 145 ppm (**b**), and D_2_O (**c**) (n = 3, *p* = 0.95).

High-sensitivity dynamic light scattering (DLS) technology (Figure 1) was used to determine the hydrodynamic diameter of water clusters in the range of 0.1–10^4^ nm.

Figure 1 shows the average values of hydrodynamic diameters of water clusters on the differential distribution curve corresponding to three modes. The average diameter value on the bimodal distribution in intensity units from 105 to 7000 nm in a deuterium-depleted water sample corresponds to d_I_ = 3500 nm (Figure 1a). Wide peaks with a satisfactory resolution at d_I_ = 3, 30, and 1050 nm are typical for a sample of ultrapure water with a natural content of the heavy hydrogen isotope (Figure 1b). Figure 1c shows a narrow particle size distribution. A large proportion of clusters were distributed in the d_I_ range: 70–600 nm. The position of the third maximum in the D_2_O sample is shifted to the micron region (d_I_ > 10^4^ nm) (Figure 1c). Thus, the analysis of fluctuations in the intensity of laser light in water with different deuterium contents allows characterizing the unique distribution of water clusters.

As we noted before, variations in the D/H ratio in water change the properties of aqueous solutions. This makes it possible to use the water isotopologues as a “tool” for controlling substance dissolution. Therefore, the objects in this study of the dissolution rate of poorly soluble medicinal substances are deuterium depleted water and water with a natural content of (2)H (deuterium).

### 2.2. Selection of the Investigating Method for Poorly Soluble Drugs’ Dissolution

**Figure 2:**
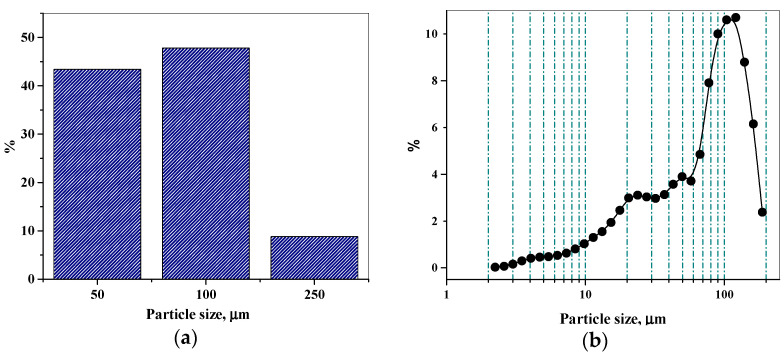
The particle-size distribution (PSD) of the topiramate powder based on data obtained by optical microscopy (**a**) and powder dispersed in fluid 0.5%—by laser diffraction analyzer (**b**).

A characteristic property of dispersion systems formed during the preparation of aqueous solutions of poorly soluble substances is the phase interface. The analysis of the distribution of dispersion-phase particle sizes of the poorly soluble in water (1:30–100 mL) substance of topiramate (central nervous system agents) shows that about 8% of the particles have a size exceeding 200 µm; a large proportion (48%) is the size group of 100 µm; and size groups below 50 µm are found at 44%. The specific surface area and volume concentration values were 0.076 (cm^2^_·_g^−1^) and 0.055%, respectively (Figure 2).

Thus, the aqueous solution of topiramate belongs to the coarse particle dispersion system. The particles of the dissolved substance retain all the properties of the phase, so its solution is a heterogeneous system [39,40,41]. The heterogeneous dissolution process of substances is limited by diffusion, adsorption, and desorption according to Fick’s law and the dissolution of the Nernst-Shchukarev equation using the following formula [42]:

dC/dt = κS (C_saturated_ − C_t_) (1)

k = D·S/δ·V(2)

where dC/dt is the dissolution rate; k is the rate constant depending on the temperature and nature of substances; C_saturated_ is the concentration of the saturated solution; Ct is the concentration of the solution at a given moment; S is the surface of the solid; D is the diffusion coefficient; δ is the thickness of the diffusion layer; when D, S, δ and V are constant, the dissolution rate constant k is a constant value.

Various methods of analysis are used for kinetic studies of dispersion systems [43,44]. The advantages of the method of laser diffraction of light over other similar methods (nephelometry, turbidimetry) made it possible to apply it to kinetic studies of dissolution. The method of laser diffraction of light is based on fundamental principles: the angular dependence of scattered laser light on the size and optical properties of particles; wide dynamic range; the universality of application; analysis of the sample as a whole, not its individual parts; non-destructivity.

**Figure 3:**
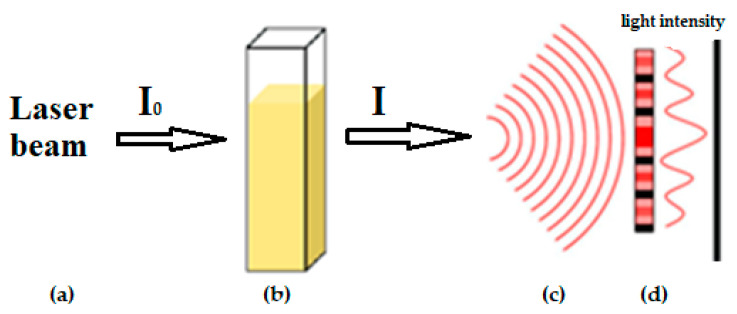
Simplified LALLS scheme for dissolution kinetics investigation: (**a**)—He-Ne laser (632.8 nm); (**b**)—measurement cell with the powder dispersed in fluid; (**c**)—diffraction pattern; (**d**)—detector.

The laser diffraction method is based on the Mie theory and registration at the time of the light scattering indicatrix arising from the interaction of electromagnetic radiation with particles of the dispersed phase [45]. The decrease in the dispersion of the sample over time results in a change in the angle distribution of the scattering intensity (low-angle laser light scattering, LALLS):

πd/λ(3)

where λ is the wavelength of the electromagnetic radiation, and d is the particle size [46]. Mathematically, laser obscuration (LO) may be presented by the following form:

LO= 1 − I/I_0_ × 100% (4)

where I is the light intensity measured by the detector when the particle is inside the measurement cell, and I_0_ is the light intensity measured by the detector with no particle in the measurement cell (Figure 3).

**Figure 4:**
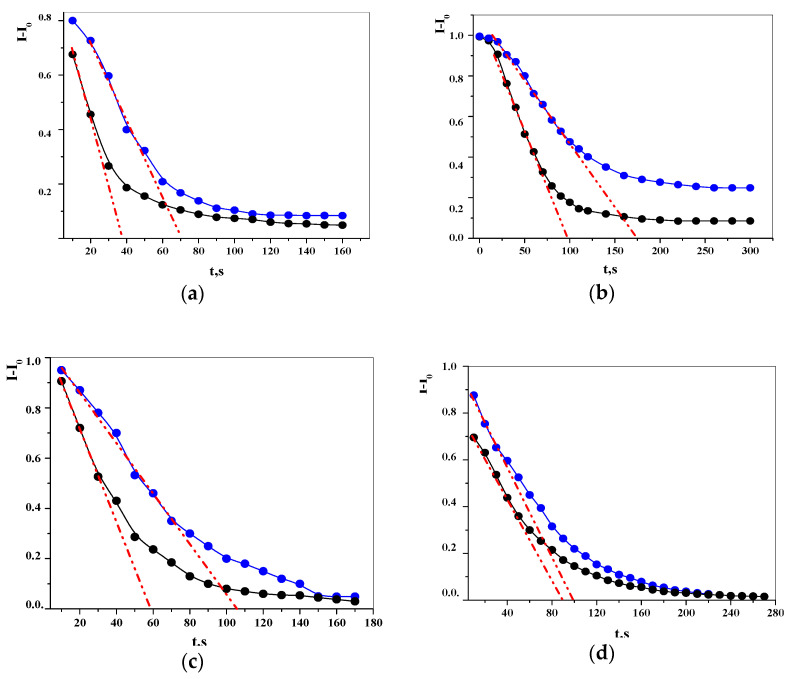
Exponential curves for the dissolution of poorly soluble drugs in different water isotopologies (black—deuterium-depleted water; blue—ultrapure water; red—tangent to the curves) by laser scattering (n = 6, P = 0.95): (**a**)—moxifloxacin hydrochloride, (**b**)—bendazol hydrochloride, (**c**)—topiramate, (**d**)—taurine. The primary powder concentration in fluid is 1 × 10^−2^ mg·mL^−1^.

Thus, using the advantages of the LALLS method, i.e., continuous monitoring of the dissolution process by registering LO values over time, high repeatability and speed of the analysis, we applied this method to study the kinetic isotope effect of the solvent in solutions of poorly soluble medicinal substances. The kinetics of the dissolution of pharmaceutical substances in water (Figure 4) is the two-step process: the sharp LO decrease from the onset of dissolution (first step) is succeeded by a gradual decrease in the LO values to reach the plateau (second step). As we have shown earlier for moxifloxacin hydrochloride and topiramate [47], the dissolution process obeys first-order kinetics and the rate constant can be calculated from the slope tangent in the coordinates «ln (LO_0_/LO) − t».

The development and further implementation of a new analytical procedure are to accompanied by validation tests, which should demonstrate that the analytical procedure is suitable for its intended purpose [48].

### 2.3. Validation of the Proposed Method

The proposed technique for assessing the dissolution rate of poorly soluble drugs of the fluoroquinolone group by LALLS in water isotopologues has been validated in studies on repeatability, intermediate precision (within-lab reproducibility), linearity, and range parameters [49].

#### 2.3.1. Repeatability

The repeatability of the technique was assessed under the conditions when six independent measurement results were obtained by the same method, in the same laboratory, by the same operator, using the same equipment, within a short time elapsed between measurements. By example, to assess the validation characteristics of the method, we present the results obtained for moxifloxacin hydrochloride as a model object.

The repeatability of the technique is represented by the values of standard deviation, coefficient of variation, and confidence interval (Table 2) [50,51].

Based on the assessment of the repeatability parameter, the intermediate precision studies were carried out.

#### 2.3.2. Intermediate Precision (Within-Lab Reproducibility)

Reproducibility is the precision estimate obtained when a series of measurements is made under more variable conditions, i.e., the same laser scattering technique for dissolution on identical test items of poorly soluble moxifloxacin hydrochloride used by different operators with different equipment in different facilities at different times (Table 3).

#### 2.3.3. Linearity and Range

**Figure 5:**
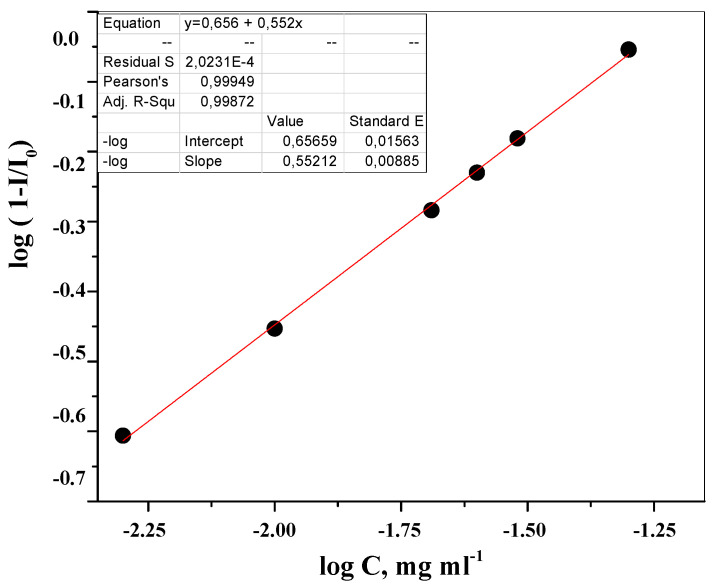
Linearity graph of moxifloxacin hydrochloride dissolution kinetics by LALLS.

The linearity, as a direct dependence of the recorded LOT signal within the analytical region, was confirmed by the creation of model weighed portions of moxifloxacin hydrochloride using the laser scattering technique. The linearity was assessed as a directly proportional signal (response) to the concentration “log (1 − I/I_0_) − log C, mg·mL^−1^” (Figure 5).

The linearity test showed a linear response for concentrations from 0.5 × 10^−2^ to 5.0 × 10^−2^ mg×mL^−1^.

### 2.4. Kinetic Solvent Isotope Effects in the Dissolution of Poorly Soluble Drugs

The analysis of the exponential curves revealed differences in the dissolution of substances in water depending on the content of the heavy hydrogen isotope: the first stage of dissolution of a substance in water depleted by a heavy isotope proceeds, on average, 1.5–3 times faster than in ultrapure water (UPW). A quantitative measure of the kinetic isotope effect is the ratio of the rate constant of dissolution in deuterium-depleted water to the rate constant of dissolution in water with a natural deuterium content (k_H_/k_D_) (Table 4).

The correlation was found between the lipophilicity of the substance and the magnitude of the kinetic isotope effect of the solvent.

The observed changes in the dissolution rate constants of poorly soluble drugs, as a result of the isotopic substitution of deuterium with protium in the water molecule, indicate the implementation of the kinetic isotope effect (KIEs) of the solvent.

The results of the implementation of the kinetic isotope effect in aqueous solutions of medicinal substances show the dependence of KIEs on the hydrophobicity of molecules: the solubility in deuterium-depleted water, in comparison with water of natural isotopic composition, increases for substances with large values of the octanol/water partition coefficient.

## 3. Discussion

The heterolytic process of dissolution of the dispersion-phase particles is accompanied by the formation of an activated complex. In this case, the solvate shells of the initial state of the dispersion-phase particles and the shells of the activated complex undergo a significant rearrangement. Therefore, it is possible to assess the influence of the nature of the solvent on the course of the dissolution process. This confirms the important role of variations in the isotopic composition of the solvent (water) in the kinetic changes of dissolution [53,54].

Replacing an atom of one of element with its isotope leads to a change in the reactivity of molecules, which is expressed in a change in the reaction rate constant, i.e., manifestation of the kinetic isotope effect (KIE). This is due to an increase in the energy required to break the bond, for example, R–D versus R–H [55,56]. That is why the reaction rate with the participation of a deuterated compound is lower than for its counterpart (k_H_ > k_D_). Kinetic isotope effect, expressed as the ratio of the reaction rate constants of the protium and deuterated compounds k_H_/k_D_, usually ranges from 1 to 7, but may be higher [57]. Deuterium-modified bioisosteres are characterized by invariability of biological activity and optimization of pharmacokinetics.

The kinetic isotope effect in drug chemistry has made it possible to synthesize deuterated substances [58]. The improvement in pharmacokinetic characteristics as a result of deuteration allows several times the therapeutic dose compared to the non-deuterated analogue.

Thus taking into account the numerous data on the KIE of pharmaceutical substances in waters with different D/H ratios, it can be predicted that deuterium will become a medico-chemical instrument [59].

## 4. Materials and Methods

### 4.1. Water Samples

The dissolution rate kinetics of poorly soluble drugs were researched in water solvents: ultrapure water (UPW) (>18 MΩ × cm^−1^ at 25 °C, TOC ≤ 5 ppb, Merck Millipore) with natural content of stable hydrogen (2)H isotope ~150 ppm; deuterium-depleted water (≤1, ppm deuterium oxide) obtained by means of liquid hydrogen low temperature vacuum rectification. Chemical purity: 99.5% for H_2_O (Merck, Darmstadt, Germany); deuterium oxide (99.9 atom % D, ALDRICH, Darmstadt, Germany).

The water samples were filtered through a submicron inert membrane filter (Millex-GV Filter with a 0.22 µm pore size hydrophilic PVDF membrane, Merck Millipore, Watford, UK).

### 4.2. Drug Samples

**Figure 6:**
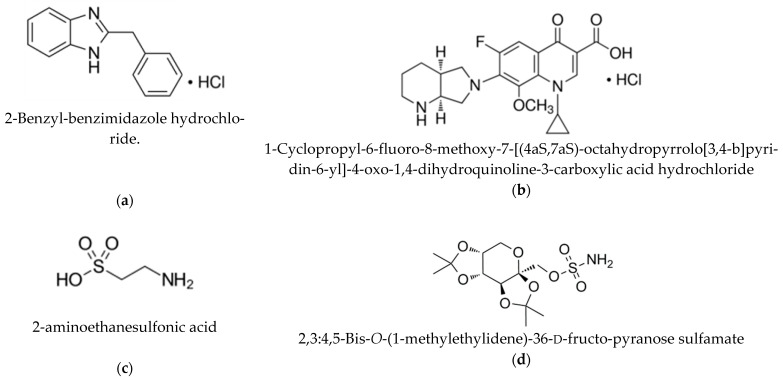
Chemical structures of poorly soluble drugs: (**a**)—bendazol hydrochloride, (**b**)—moxifloxacin hydrochloride, (**c**)—taurine, (**d**)–topiramate.

There were utilized ingredients of various pharmaceutical and chemical groups, which are characterized by a specific solubility in water: moxifloxacin hydrochloride is a fourth-generation synthetic fluoroquinolone antibacterial (Second Pharma Co., Ltd., Shaoxing Shi, Zhejiang Sheng, China); bendazol hydrochloride—adjuvant, immunologic (Second Pharma Co., Ltd., China series P111106); topiramate—sulphate fructopyranose derivative, neuroprotective agent, anticonvulsant (≥98%, Xian Bodigard Pharmaceutical Co., Ltd., China); taurine (≥99%, Sigma, Darmstadt, Germany) (Figure 6) (Table 5).

### 4.3. Granulometric Analysis

The size and shape of the substance particles were determined by optical microscopy method (microscope Altami BIO 2, Russia) with a 10-fold increase. The sample was applied to the slide and evenly distributed over the entire surface. The particles were observed in 10 separate fields of vision.

The granulometric analysis of the samples was carried out by the method of low-angle laser light scattering (LALLS) using a Mastersizer 3600 (Malvern Panalytical, UK).

### 4.4. Dynamic Light Scattering Method

The method of dynamic light scattering (DLS, photon correlation spectroscopy, quasi-elastic light scattering), based on the analysis of the Brownian motion of dispersion-phase particles and on the occurrence of fluctuations in the local particle concentration, was used to determine the hydrodynamic diameter of clusters in the range of 0.1–10^4^ nm in water samples with different (2)H (deuterium) contents.

### 4.5. Research Method for Evaluation of Poorly Soluble Drugs’ Dissolution Rate by Laser Light Scattering

The intensity of laser light scattering over time was determined using a low-angle laser particle dispersion meter, the Particle Sizer (Malvern Instruments, Malvern, UK), with a scanning wavelength of λ = 632 nm and capacitive cell (V = 3 mL) equipped with a mechanical stirrer.

The radiation scattered by the dispersion-phase particles was detected at different angles using a highly sensitive multi-element detector—photodiode array. Three repeated measurements were made on each aliquot using 30 s as the measurement time, which is equivalent to 30,000 individual light scattering measurements [60]. The measurement of laser obscuration time (LOT) was started while adding water to the cell and continued with intervals of 10 s up to the complete dissolution of the substance which was recorded up to the end of change in the time of the examined laser obscuration parameter.

### 4.6. Statistical Data Processing

All statistical data processing was performed using Student’s *t*-test, as well as using one-way analysis of variance (ANOVA) in the Origin Pro software. The differences were considered statistically significant at *p* < 0.05.

## 5. Conclusions

The article presents the results of studies on the influence of the kinetic isotope effect of a solvent-water-on increasing the solubility and dissolution rate constants of poorly soluble drugs using laser diffraction spectroscopy. The conducted studies allow evaluating the important role of varying the isotopic composition of water in correcting the solubilization characteristics of poorly soluble drugs in order to improve their pharmacokinetic characteristics. The results of the studies can be successfully implemented in pharmaceutical practice to overcome the poor solubility of medicinal substances of classes II and IV, according to the biopharmaceutical classification system (BCS), in water for pharmaceutical purposes by performing its preliminary and safe isotopic modification.

## Figures and Tables

**Table 1:** Data on laser light scattering in water samples with different contents of stable hydrogen isotopes.

LALLS Data	Values		
	Deuterium-Depleted Water (ddw), ≤1 ppm D/H	Ultrapure Water (UPW), ~150 ppm D/H	Deuterium Oxide, D_2_O 99.9%
Volume concentration of water clusters, vc (%)	0.004	0.017	0.003
Laser obscuration (λ = 632.8 nm)	0.17	0.43	0.61

**Table 2:** Assessment of the repeatability of the analytical technique (n = 6; *p* = 0.95).

$\bar{k}$, s^−1^	SD	S^2^	RSD, %	$\left(\bar{k}\pm \Delta \bar{k}\right)$, s^−1^
0.058	3.1 × 10^−3^	9.6 × 10^−6^	5.3	0.058 ± 0.003

**Table 3:** Assessment of the intermediate precision of the analytical technique (n = 6; *p* = 0.95).

$\bar{k}$, s^−1^	SD	S^2^	RSD, %	$\left(\bar{k}\pm \Delta \bar{k}\right)$, s^−1^	$\bar{\varepsilon }$, %
0.057	4.7 × 10^−3^	2.3 × 10^−5^	8.3	0.057 ± 0.002	4.1

**Table 4:** The rate and kinetic solvent isotope effects (KIEs) of poorly soluble drugs dissolution in different water isotopologues.

Name of Poorly Soluble Drug	$\left(\bar{k}\pm \Delta \bar{k}\right)$, s^−1^		$\frac{k_{H}}{k_{D}}$	log P_oct/water_ [52]
	Water, Deuterium-Depleted (ddw)	Ultrapure Water (UPW)		
Bendazol hydrochloride	(1.92 ± 0.07) × 10^−2^	(0.84 ± 0.04) × 10^−2^	2.3	2.8
Moxifloxacin hydrochloride	(5.50 ± 0.003) × 10^−2^	(1.57 ± 0.004) × 10^−2^	3.5	2.9
Taurin	(1.99 ± 0.04) × 10^−2^	(1.71 ± 0.02) × 10^−2^	1.2	−1.3
Topiramate	(2.89 ± 0.001) × 10^−2^	(1.83 ± 0.001) × 10^−2^	1.6	1.3

**Table 5:** Pharmacopeia solubility of drug samples.

Drug Samples	Solubility in Water (Descriptive Term, Approximate Volume of Solvent in Milliliters per Gram of Solute) *
bendazol hydrochloride	sparingly soluble (from 30 to 100)
moxifloxacin hydrochloride	sparingly soluble (from 30 to 100)
taurine	soluble (from 10 to 30)
topiramate	sparingly soluble (from 30 to 100)

* European Pharmacopoeia (Ph. Eur.) 10th Edition.

## Data Availability

The data presented in this study are available on request from the corresponding author.

## Sample Availability

Samples of the compounds are available from the authors.
